# Supplementary material for: A link between aging and persistence
Source: Antimicrob Agents Chemother. 2025 Feb 21;69(4):e01313-24. doi: 10.1128/aac.01313-24 (PMC11963536; doi:10.1128/aac.01313-24)
Supplement: Supplemental material — Supplemental methods, Figures S1 to S4, and Table S1. [file aac.01313-24-s0001.pdf]

Supplementary Information

A link between aging and persistence

A. M. Proenca[1,2#], C. U. Rang[3], L. Chao[c#]

AFFILIATIONS

[1] Immunology and Microbiology Laboratory, School of Health and Life Sciences, Pontifical Catholic University of Rio Grande do Sul; Porto Alegre, RS, Brazil.

[2] Institute of Biology, Freie Universität Berlin; Berlin, Germany.

[3] Section of Ecology, Behavior and Evolution, Division of Biological Sciences, University of California, San Diego; La Jolla, CA, USA.

[#] Address correspondence to A. M. Proenca, aproenca@zedat.fu-berlin.de, and L. Chao, lchao@ucsd.edu

SUPPLEMENTARY METHODS

Bacterial aging model

Aging and rejuvenation contribute to the heterogeneity of growth states found within clonal bacterial populations. To frame this age structuring, we can apply a population genetics model that describes bacterial growth and division across generations (*35*). For this, let us imagine that an *E. coli* cell is born free of intracellular damage ($k_0 = 0$). Over its lifetime, it accumulates damage at a rate λ:

$$k(t) = k_0 + \lambda t \tag{1}$$

This cell must also accumulate a certain amount of internal product in order to divide, similarly to an "adder" model (*52*). A damage-free cell would reach this checkpoint at time Π, the minimum possible doubling time (T_0):

$$\Pi = (1 - k_0)T_0 - (\lambda/2)T_0^2 \tag{2}$$

Realistically, however, even cells cultured in benign environmental conditions accumulate intrinsic damage at a rate $\lambda > 0$ min^{-1}, which slows down the accumulation of internal product and leads to longer doubling times (T_0). Assuming that λ is linear, the cell will divide with a damage load D_0:

$$D_0 = k_0 + \lambda T_0 \tag{3}$$

Upon division, this damage load is partitioned between the daughters with a certain degree of asymmetry (*a*). We define that *a* represents the fraction of D_0 inherited by the new daughter, such that *a* = 0 when the old daughter receives the full maternal load, and *a* = 0.5 for a symmetric division:

$$k_1 = D_0 a = (k_0 + \lambda T_0)a \tag{4}$$

$$k_2 = D_0(1 - a) = (k_0 + \lambda T_0)(1 - a) \tag{5}$$

The amount of damage inherited by new (k_1) and old (k_2) daughters, along with λ, will determine their respective doubling times (T_1 and T_2) at division:

$$T_i = \frac{\left\{(1 - k_i) - \sqrt{(1 - k_i)^2 - 2\Pi\lambda}\right\}}{\lambda} \tag{6}$$

The model thus allows us to estimate the doubling times of new and old daughters based on the initial damage load of the mother. To examine the core predictions generated from this mother, shown in Fig. 1, we used values for the parameters Π, λ, and *a* obtained from previous experiments (*30*). For an unstressed population, we assumed that Π = 18.5 min, λ = 0.0022 min^{-1}. For a population facing high levels of stress, we increased damage accumulation rates to λ = 0.0089 min^{-1}. Asymmetry values were assumed to vary with λ, as

$$a = 0.1007 \ln(\lambda) + 0.9531 \tag{7}$$

according to experimental observations (*34*). If we propagate lineages inheriting either new or old poles forward in time, we obtain an interesting prediction. Provided that λ remains low, a lineage of new pole cells would reach a point where $k_0 = k_1$, and therefore $T_0 = T_1$ (Fig. 1B). The same occurs if we follow a lineage of old pole cells, which reach a point where $k_0 = k_2$ and $T_0 = T_2$. When plotting maternal doubling times against that of each daughter (Fig. 1B), these equilibrium points are represented as the intersection between model predictions and the identity line.

As λ increases however, populations reach a threshold where new daughter lineages can still reach the $T_0 = T_1$ equilibrium, but the old daughter lineage can no longer stabilize. As a result, T_2 assumed increasingly larger values, until the lineage ceases to divide (Fig. 1C).

SUPPLEMENTARY FIGURES

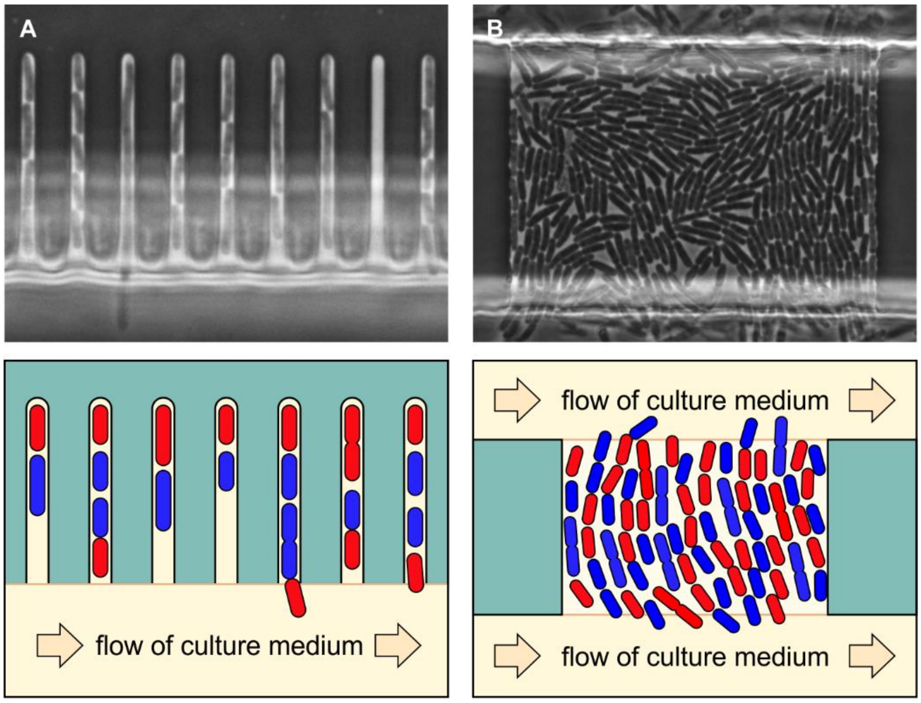

Fig. S1 Microfluidic devices under phase contrast microscopy. (A) The mother machine consists of growth wells with one closed end (1.25 × 30 × 1 µm), which trap the old daughter lineage for the duration of the experiments. **(B)** The daughter device consists of large growth chambers (40 × 50 × 0.95 µm) flanked by flow channels, accommodating a population with no bias towards either new or old daughters.

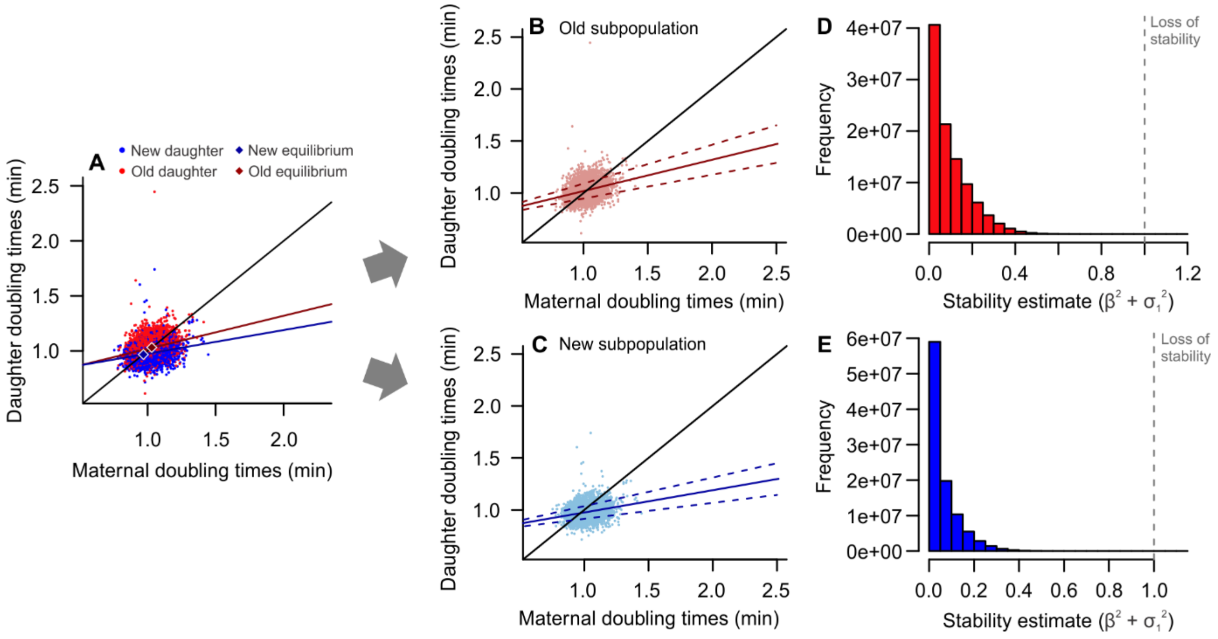

Fig. S2 Stability of growth equilibria in exponential populations. (A) Individual data points used in Fig. 2B are presented without binning. The predicted points of equilibrium for new and old subpopulations are indicated through linear regressions between mother and daughter doubling times. **(B)** Old daughters showed a positive correlation with maternal doubling times ($\beta = 0.313$, $R^2 = 0.092$, $p < 0.001$; solid red line). The stochasticity present in the system is shown as dashed lines, representing the standard deviation of all slopes between mother and daughters ($\sigma_1 = 0.072$). Because $\beta^2 + \sigma_1^2 = 0.103$, the condition $\beta^2 + \sigma_1^2 < 1$ is satisfied and this equilibrium is stable. **(C)** The same pattern was observed for new daughters ($\beta = 0.214$, $R^2 = 0.062$, $p < 0.001$; solid blue line), with $\sigma_1 = 0.061$. Because $\beta^2 + \sigma_1^2 = 0.050$, the equilibrium is stable. **(D and E)** To determine whether such levels of asymmetry and stochasticity could yield persisters given a larger sample size, we simulated populations of 10^5 cell lineages propagated for 30 generations with a given β and σ_1. This was performed for new and old subpopulations separately, with a 1,000x bootstrap for each. The histograms show the distributions of stability estimates for all resulting old **(D)** and new **(E)** lineages.

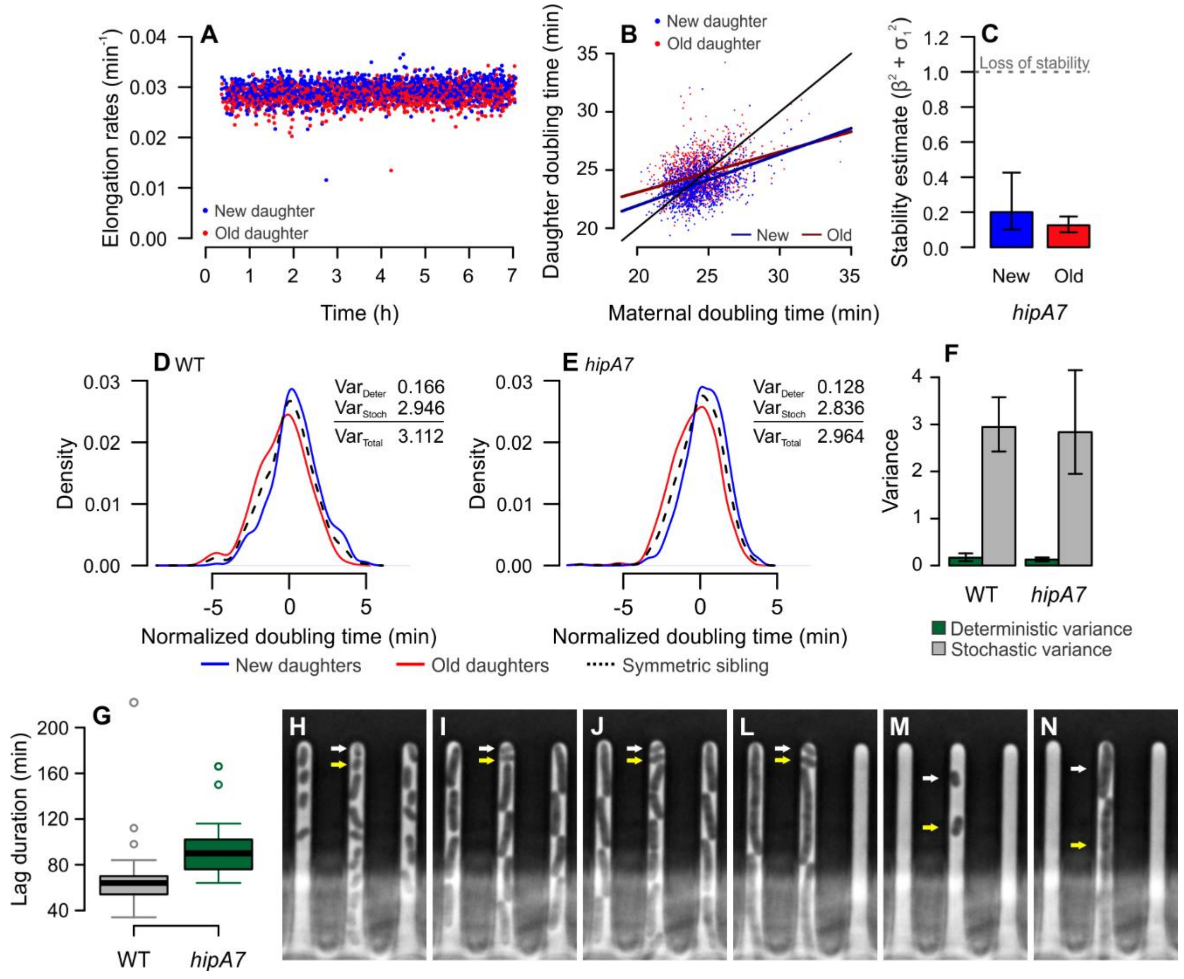

Fig. S3 ***hipA7*** **exhibits cellular aging and stable growth. (A)** The high-persistence mutant had stable elongation rates over time, with new daughters displaying faster growth (0.029 ± 0.002 min^{-1}, n = 1,565 cells) than old daughters (0.028 ± 0.002 min^{-1}, n = 1,505 cells; one-tailed t test, t = 13.37, df = 3,063.1, $p < 0.001$). **(B)** Maternal doubling times showed positive correlation with new ($\beta = 0.443$, $p < 0.001$, $R^2 = 0.148$) and old ($\beta = 0.349$, $p < 0.001$, $R^2 = 0.095$) subpopulations*. Slopes intersecting the identity line suggested points of stable physiology. **(C)** These regressions were used to estimate the stability of equilibrium points according to the slope (β) and stochasticity (σ_1) for each subpopulation. Both new and old lineages had stable equilibria, suggesting continuous growth and replication despite stochastic processes. Error bars = 95% CI. **(D to F)** Partitioning of doubling time variance into deterministic and stochastic components. The distance between distributions provides an estimate of deterministic variance, whereas the mean variance of new and old doubling times distributions estimates the stochastic variance. Compared to wild-type *E. coli*, the *hipA7* mutant did not have higher levels of stochasticity on its growth heterogeneity. Error bars = 95% CI. **(G)** The main physiological distinction between strains was the longer lag phase of *hipA7* (one-way ANOVA, F = 64.112, $p < 0.001$). **(H to N)** When cells were exposed to 100 µg/ml Ampicillin shortly after loading, lag phase individuals (arrows) persisted the antibiotic treatment. Time-lapse images are shown in 100 min intervals. The effects of Ampicillin can be observed on (J and L), leading to lysis of growing cells. After 5h, once the antibiotic was removed, persisters started growing (**N**).

* Two points in **B** (daughter doubling times of 51.71 and 60.03 min) are not shown due to plot proportions, but appear as elongation rates in **A**.

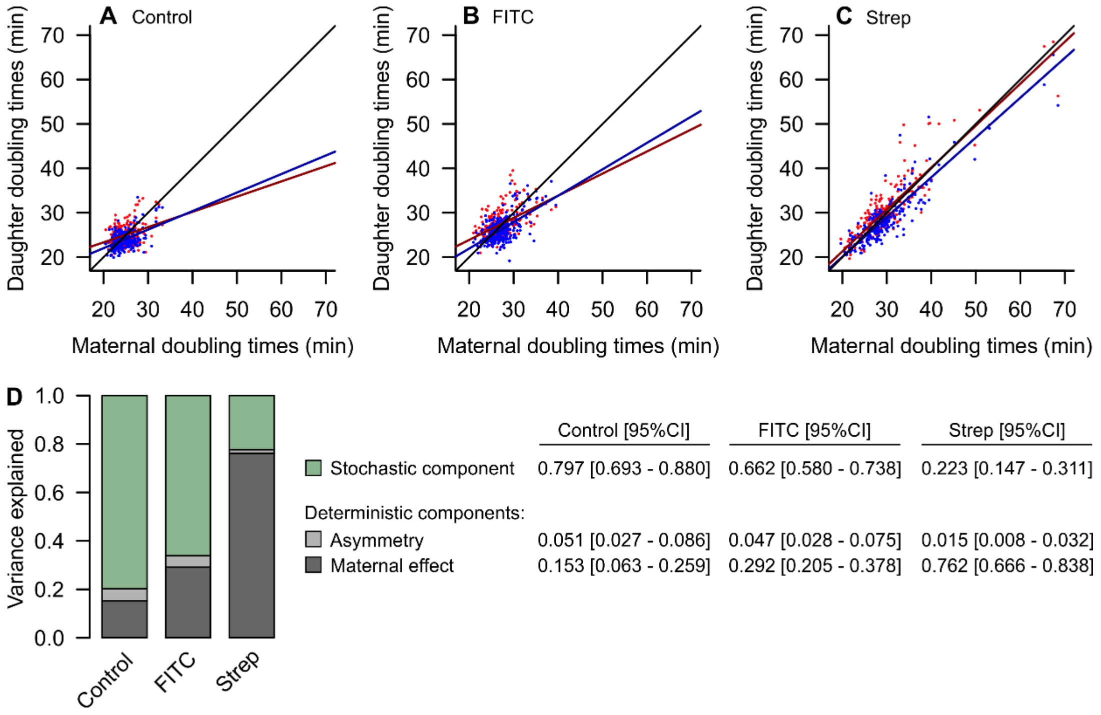

Fig. S4 Deterministic and stochastic components of the variance for populations under oxidative stress. (A to C) Phase planes showing the doubling times of new and old daughters as a function of maternal doubling times. **(D)** The total variance of these doubling times was decomposed into its deterministic (asymmetry and maternal effects) and stochastic components through the sums of squared deviations method (see Methods for details). Contrary to the analysis presented in Fig. S3D-F, which compared absolute variance, this estimate considers the relative contribution of each component. Populations exposed to photo-oxidation and Streptomycin showed increasingly more deterministic variability, likely driven by the inheritance of larger damage loads from a mother cell.

Table S1. Equilibrium parameters for exponential and stationary phase populations. The parameters were obtained from linear models between each daughter subpopulation and maternal doubling times (Fig. 4C), and are presented as mean [95% confidence intervals]. For each set of parameters, we simulated the formation of persisters over 30 generations in a population of 100,000 lineages, starting from the predicted equilibrium at t = 1. Each simulation was repeated 1,000 times.

	**Exponential phase**		**Stationary phase**	
	**New lineages**	**Old lineages**	**New lineages**	**Old lineages**
Slope (β)	0.325 [0.164 - 0.481]	0.345 [0.197 - 0.503]	0.447 [0.324-0.601]	0.503 [0.288 - 0.772]
y-axis intersect (b)	14.369 [10.580 - 18.268]	15.663 [11.864 - 19.219]	55.686 [41.250 - 67.805]	60.342 [33.899 - 82.678]
Stochasticity (σ_1)	0.054 [0.046 - 0.062]	0.062 [0.054 - 0.068]	0.414 [0.336 - 0.495]	0.857 [0.474 - 1.168]
Predicted equilibrium (min)	21.291 [20.360 - 21.933]	23.915 [23.515 - 24.304]	100.646 [92.488 - 111.616]	121.309 [107.371 - 156.394]
Stability ($\beta^2 + \sigma_1^2$)	0.109 [0.029 - 0.235]	0.123 [0.042 - 0.258]	0.371 [0.217 - 0.606]	0.988 [0.307 - 1.960]
Persistence simulation				
# of populations containing persisters (out of 1,000)	25	35	1,000	1,000
Average # of persisters in a population (out of 100,000 lineages)	0.025	0.035	205	28,477
Persister frequency	2.50×10^{-7}	3.50×10^{-7}	2.05×10^{-3}	2.85×10^{-1}
